# Primary extrahepatic alveolar echinococcosis of the lumbar spine and the psoas muscle

**DOI:** 10.1186/1476-0711-10-13

**Published:** 2011-04-15

**Authors:** Manuel Nell, Rainer H Burgkart, Guntmar Gradl, Rüdiger von Eisenhart-Rothe, Christoph Schaeffeler, Dennis Trappe, Clarissa Prazeres da Costa, Reiner Gradinger, Chlodwig Kirchhoff

**Affiliations:** 1 Department of Orthopaedics and Traumatology, Technische Universität München, Klinikum Rechts der Isar, Ismaninger Str. 22, D-81675 Munich, Germany; 2 Department of Radiology, Technische Universität München, Klinikum Rechts der Isar, Ismaninger Str. 22, D-81675 Munich, Germany; 3 German Consiliary Labaratory for Echinococcosis, Institute of Hygiene and Microbiology, University of Würzburg, Josef-Schneider-Strasse 2, 97080 Würzburg, Germany; 4 Institute for Medical Microbiology, Immunology and Hygiene, Technische Universität München, Klinikum Rechts der Isar, Trogerstr. 30, 81675 Munich, Germany

## Abstract

Alveolar echinococcosis (AE) of human being caused by Echinococcus multilocularis is a rare but important zoonosis especially in tempered zones of middle Europe and Northern America with endemic character in many countries. Due to the long incubation period, various clinical manifestations, critical prognosis, and outcome AE presents a serious and severe disease. The primary focus of infection is usually the liver. Although secondary affection of visceral organs is possible extrahepatic AE is highly uncommon. Moreover, the involvement of bone and muscle presents with an even lower incidence. In the literature numerous cases on hepatic AE have been reported. However, extrahepatic AE involving bones and/or muscles was described very rarely. We report a case of an 80-year-old man with primary extrahepatic alveolar Echinococcosis of the lumbar spine and the psoas muscle. The etiology, diagnosis, differential diagnoses, treatment options and outcome of this rare disease are discussed in context with the current literature.

## Introduction

Two types of Echinococcus species are known - Echinococcus granulosus (EG) and Echinococcus multilocularis (EM). EG causes cystic Echinococcosis (CE) while EM causes alveolar Echinococcosis (AE) [1-3]. AE is one of the most pathogenic zoonoses in the northern hemisphere with an annual incidence of 0.03 to 1.2 per 100.000 inhabitants [4,5]. In Europe EM is endemic in Belgium, Luxembourg, France, Switzerland, Liechtenstein, Germany, Austria, Italy, Poland, and the Czech Republic. Latest epidemiology research on EM in Europe revealed additional endemic regions within urban and suburban areas. This seems to be due to increasing prevalence of EM in foxes (Vulpes vulpes), the primary host [4,6]. Besides foxes, dogs and cats, also domestic pigs might be affected by EM [3].

Usually, the primary infection site of AE is the liver. However, it can spread into extrahepatic structures and metastasize like a tumorous disease even to remote organs such as brain and heart. The incubation time ranges from five up to fifteen years [1,7]. Owing to its infiltrating and metastasizing character, AE is clinically staged according to the Primary-tumor-Nodes-Metastasis-system (PNM)-system. It is based on the extent of the hepatic lesion, the affection of adjacent organs as well as the number and extent of metastases [1] (see table 1). AE usually metastasizes to pancreas, spleen, retroperitoneum, lung, brain, but also to bone and soft tissue [8]. Gottstein et al. described an occurrence of metastasis in up to 20%, especially in lung and brain [9]. While pulmonary metastases occur in 7 to 20%, cerebral metastases are only described in 1 to 3% [1,5]. Osseous affection is uncommon, occurring in up to 1% of all cases [10]. To the best of our knowledge only 18 cases of osseous AE have been reported so far [11,12]. Primary extrahepatic manifestation of AE seems to be absolutely unusual and extremely rare.

**Table 1:** The PNM classification of alveolar echinococcosis

P	Hepatic localization of the parasite
PX	Primary tumor cannot be assessed
P0	No detectable tumor in the liver
P1	Peripheral lesions without proximal vascular and/or biliary involvement
P2	Central lesions with proximal vascular and/or biliary involvement of one lobe *(a)*
P3	Central lesions with hilar vascular or biliary involvement of both lobes and/or with involvement of two hepatic veins
P4	Any liver lesion with extension along the vessels *(b) *and the biliary tree
N	Extra-hepatic involvement of neighboring organs [diaphragm, lung, pleura, pericardium, heart, gastric and duodenal wall, adrenal glands, peritoneum, retroperitoneum, parietal wall (muscles, skin, bone), pancreas, regional lymph nodes, liver ligaments, kidney]
NX	Not evaluable
N0	No regional involvement
N1	Regional involvement of contiguous organs or tissues
M	The absence or presence of distant Metastasis [lung, distant lymph nodes, spleen, CNS, orbita, bone, skin, muscle, kidney, distant peritoneum and retroperitoneum]
MX	Not completely evaluated
M0	No metastasis *(c)*
M1	Metastasis

*(a) *For classification, the plane projecting between the bed of the gall bladder and the inferior vena cava divides the liver in two lobes

*(b) *Vessels mean inferior vena cava, portal vein and arteries

*(c) Chest *X-ray and cerebral CT negative

In this context we present the case of a primary extrahepatic AE, affecting the lumbar spine and the psoas muscle. We discuss the etiology, diagnosis, and therapy of this rare lesion based on the current literature.

### Case history

An 80-year-old man presented with sudden onset of right upper and lower quadrant abdominal pain at an academic department for surgery in October 2009. In addition, he suffered from relapsing nausea and vomiting, also describing recurrent low-grade fever a few days before and subjective feeling of swelling of the right upper abdomen. Written informed consent was obtained from the patient for publication of this case report and accompanying images. Computed tomography (CT) of the abdomen was performed revealing several cystic lesions of different size in the right psoas muscle. At that time, abscess-like formations were considered to be the most likely diagnosis. Considering this the working diagnosis initial treatment included the intravenous administration of antibiotics - Piperacillin/Tazobactam - and the percutaneous drainage of the biggest abscess under CT guidance. A follow-up CT-scan showed a diminution of the drained abscess followed by clinical recovery. Therefore, another percutaneous drainage of another, smaller abscess was performed. This led to shrinkage of the lesion and further clinical recovery. The inflammatory parameters decreased and no microorganisms were detected in the drained fluid. Subsequently the patient was discharged home.

One week later, the patient presented with recurrent abdominal pain in the right lower quadrant at the same department he had initially turned to. An abdominal ultrasound showed several obvious abscesses in the right psoas muscle. After readmission, three CT-guided suction drainages were placed in the three biggest abscesses. The microbiological analysis of the drained liquid provided evidence of Staphylococcus aureus. Hence antibiotic treatment was restarted with Piperazillin/Tazobactam. Despite continuous drainage for 7 days a follow-up CT revealed size constancy of the abscesses. Therefore, surgery was indicated and an open debridement of the abscesses in the right psoas muscle with resection of pannus was performed. The intraoperative microbiological work-up proved Staph. epidermidis and Staph. warneri. Thus, the antibiotic treatment with Piperacillin/Tacobactam was continued with higher dose rate. The patient's general condition improved and he could be discharged to rehabilitation 22 days after readmission.

**Figure 1:**
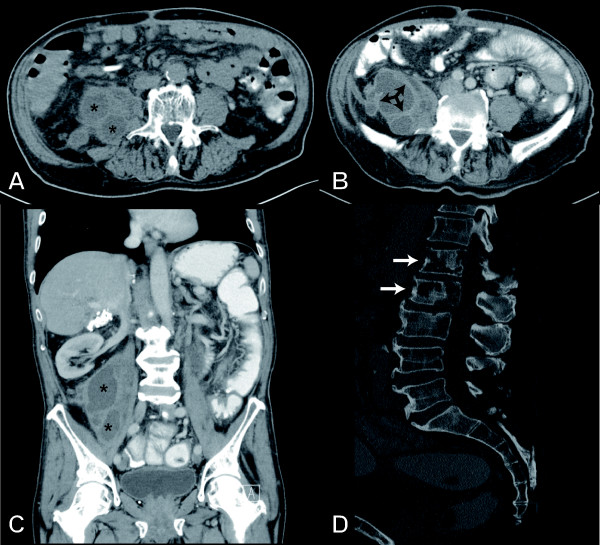
**Computed tomography scans of the abdomen and pelvis**. Non-enhanced (A) and contrast-enhanced (B) axial images show a multilobulated cystic mass in the right retroperitoneum, originating from the psoas muscle. Besides of the cystic components with fluid-like density (*) thickened septa with mild contrast enhancement (arrow) are seen. The coronal image (C) shows the extent of the mass along the psoas muscle. (D) The lumbar spine presents lytic lesions of the first and second lumbar vertebra (arrows) with partial cortical destruction.

**Figure 2:**
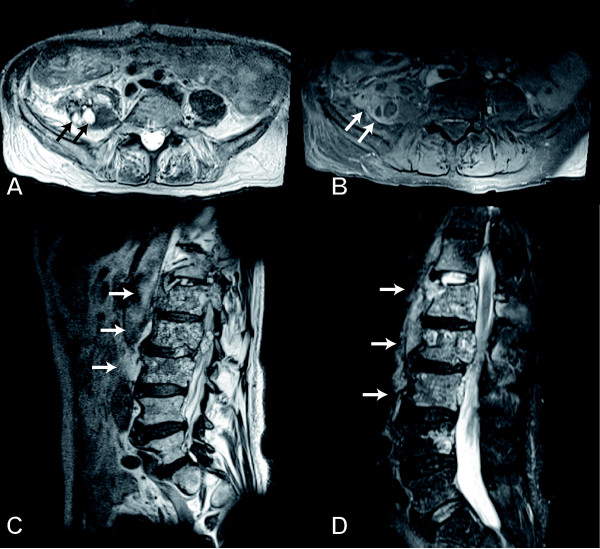
**Magnetic Resonance Imaging of the lumbar spine**. (A) The T2-weighted axial MR image shows multiple small hyperintense lesions in the right psoas muscle (arrows). The corresponding fat-suppressed T1-weighted image after gadolinium administration (B) confirmed the diagnosis of a multicystic mass and delineated the thickened, contrast-enhancing septations around the cystic components (arrow). T1-weighted image (C) and T2-weighted STIR image (D) of the lumbar spine in sagittal orientation. These images showed the bone marrow replacement within the first, second, and third lumbar vertebrae (arrows). The lesions comprise of cystic and solid components.

During rehabilitation he suffered from recurrent right lower quadrant abdominal pain and therefore he was readmitted again. After readmission a CT-scan revealed a new multilobulated cystic lesion in the right psoas muscle (see figure 1). Again a suction drainage was placed in the biggest abscess-like lesion and purulent fluid was obtained. The cultivation of the drained fluid revealed growth of Staph. lugdunensis. Therefore the antibiotic treatment was changed to Amoxicillin. Owing to drug hypersensitivity, the treatment had to be changed to Vancomycine and Zyvoxid. During hospitalization the patient developed additional pain in the upper lumbar spine from lumbar vertebrae L1 to L3 without neurological deficit. Simultaneously the general health condition of the patient worsened. A CT examination of the lumbar spine showed geographic bone destruction and inhomogeneous osteolysis of the lumbar vertebra L1 to L3 without soft tissue involvement, leading to the diagnosis of osteomyelitis (see figure 1 and 2).

**Figure 3:**
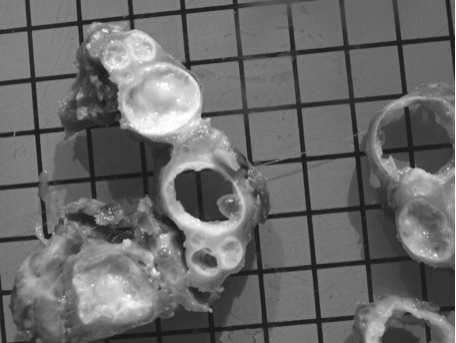
**Photograph of the resected specimen demonstrates the macroscopic appearance of the multivesicular hydatid cyst**. The typical "bunch of grapes" appearance is visible; moreover it is obvious that the hydatid cyst has three layers.

Hence, the patient was transferred to our academic department for orthopaedics and traumatology for further treatment. The range of motion of the right hip joint was painfully reduced and there was a positive psoas sign. The patient's general condition was poor and he showed a lack of appetite with consecutive weight loss. He denied nausea and vomiting, body temperature was normal. The physical examination was unremarkable. Only pressure pain to the right lower quadrant of the abdomen and percussion tenderness at the level of the lumbar vertebrae L1 to L3 was revealed. Therefore surgical revision was indicated; during surgery tubular cavities were detected macroscopically (see figure 3) and for the first time Echinococcosis was suspected. A subtotal resection of the right psoas muscle was performed due to the multitude of obvious parasitic lesions. The lumbar vertebrae L1 to L3 were removed and then filled up with polymethylmethacrylate bone cement (PMMA). The microbiological examination did not reveal any microorganisms. However the histopathological examination showed typical parasitic vesicles consistent with Echinococcosis, which were delineated by a Periodic-Acid-Schiff (PAS+) laminated layer test. The postoperative serology was highly positive for AE. In addition, polymerase chain reaction (PCR) was conducted and specific nucleic acids of EM were detected. Thus the antibiotic treatment was stopped and a continuous Benzimidazole therapy with Albendazole 400 mg twice per day was started immediately. In the course of the disease the patient's general condition improved and finally the inflammatory parameters totally declined. The patient showed great improvement regarding mobilization. A follow-up CT- and MR-examination was performed one month after the beginning of continuous Benzimidazole treatment. The images revealed no parasitic residua in the right psoas muscle, but little cystic residua in the first lumbar vertebra were depicted. Furthermore, a postoperative hematoma anterior to the psoas muscle was detected. No new parasitic cystic lesions were found. An additional cerebral CT did not show any pathological findings. Finally the patient could be discharged for rehabilitation.

## Discussion

AE represents an important endemic parasitic zoonosis in the northern hemisphere [1]. Owing to its late onset and various clinical features a certain delay until definite diagnosis is common and AE is often found incidentally.

In the present case the patient reported a long history of abdominal discomfort and pain with additional relapsing nausea, vomiting, fever, and a subjective feeling of swelling. The symptoms and clinical signs depend on the structures and/or organs affected. Therefore, the clinical presentation of AE is highly variable and not specific [13]. In hepatic affection the symptoms include cholestatic jaundice and/or abdominal pain. Further symptoms are fatigue and weight loss [2].

X-ray and CT findings of osseous AE are unspecific. Inhomogeneous osteolysis and irregular bone destruction may be seen. In the soft tissues, CT can reveal cystic masses with irregular thickened septations [14]. Magnetic resonance imaging (MRI) may show tube-like cavities/cystic lesions with a multi-vesicular morphology if soft tissue is infected. Diagnostic criteria for cystic lesions are high intensity on T2-weighted images and low intensity on T1-weighted images without contrast agent enhancement (see figure 2). Because of the contrast enhancing thickened margins and septations AE can easily be confused with abscess formations [15,16].

In this context the multiple cyst-like formations in the right psoas muscle were initially considered as bacterial psoas abscesses (BIA). Since BIA are common in the elderly population, this diagnosis seemed to be corroborated at first [17]. Moreover, antibiotic treatment and percutaneous drainage lead to a diminution of the abscess formation and improved general health status. Additionally the diagnosis of BIA was even more corroborated because of the growth of various Staph. species. According to Ricci et al. Staph. aureus is known as the most common microorganism with an incidence of over 88% in patients suffering from BIA [17,18]. In parallel to AE BIA also cause unspecific clinical symptoms [17,19-21]. The classic clinical triad of fever, back and limb pain is present in only 30% of the patients with BIA [17]. Regarding etiology BIA is related with diabetes mellitus, intravenous drug abuse, AIDS, renal failure and immunosuppression [17,22]. Vertebral osteomyelitis, septic arthritis or infectious sacroiliitis may also cause BIA [17,22].

In the present case, the patient developed lumbar back pain. The CT examination of the lumbar spine was interpreted as vertebral osteomyelitis. Therefore, therapy included antibiotic medication and percutaneous drainage. These management strategies are recommended for treatment of BIA [6,18-21,23,24]. Because of failure of the percutaneous drainage open surgery was performed in the further course.

The intraoperative aspect of tube-like cavities in the psoas muscle raised the suspicion for Echinococcosis.

Finally the presence of AE was confirmed by histopathology and laboratory setup. In addition to the radiological and macroscopic presentation the diagnosis of AE is based on serology and PCR [11]. Histopathology typically reveals parasitic vesicles delineated by a Periodic-Acid-Schiff (PAS+) laminated layer [2,10]. The periparasitic granuloma is composed of epitheloid cells lining the parasitic vesicles, macrophages, fibroblasts and myofibroblasts, giant multinucleated cells, and cells of the nonspecific immune response, surrounded by lymphocytes [2].

In the present case the postoperative serology was positive for AE. For serological diagnosis purified, recombinant or in-vitro produced E. multilocularis antigens are used, mainly Em2, Em2+ and Em18 (2). Em10 is an E. multilocularis specific recombinant antigen used for assays to confirm AE [1]. According to the literature serology has a high sensitivity of 90 to 100% and a specificity of 95 to 100% [2]. Carmena et al. described high sensitivity and specificity of serological tests ranging from 75 to 100% [25]. The differentiation between E. multilocularis and E. granulosus is possible in 80 to 95% of cases using most of the purified antigens [2]. Nevertheless serological interpretation can be difficult in disease with extrahepatic infestation and sometimes remains insufficient in differentiating AE from CE. Especially the use of total somatic antigens is considered to show a high degree of cross-reaction with other parasites [11]. In contrast, Gottstein et al. described improved immunodiagnostic with Em2+ ELISA using purified species-specific antigens ^9^. The Western blot detects serum IgG in 97% of patients infected with Echinococcus and its sensitivity is higher than of the ELISA for the detection of Echinococcosis [11]. However, it can distinguish E. multilocularis from E. granulosus in only 76% of cases.

In our patient, PCR was conducted additionally in tissue specimens resected from the psoas muscle and the lumbar vertebrae. The result was equal 100% for the presence of E. multilocularis. The aim of PCR is to detect specific nucleic acids of Echinococcus and consequently to differentiate E. multilocularis from E. granulosus. So PCR is increasingly accepted as a complementary diagnostic tool for Echinococcosis and has been used to confirm the pathology in unusual locations [1]. Scheuring et al. were successful differentiating E. multilocularis and E. granulosus in a patient with osseous and subcutaneous locations [26]. Georges et al. described two cases of extrahepatic osseous infestation where PCR was useful to diagnose AE because of limited serological interpretation [11].

Regarding medical history, vocational or part-time farming, gardening, forestry and hunting are known risk factors for a possible infection with AE [5]. 559 patients were reported to the European Echinococcosis registry from 1982 to 2000 on a voluntary basis. 61.4% of 210 registered persons presented with risk factors as listed [5]. Therapy of AE should be multidisciplinary addressing every potential organ affection. Early diagnosis is crucial for prognosis and outcome as mortality reaches up to 80% in untreated patients [27]. In Europe therapy has changed average life expectancy at time of diagnosis from 3 years in the 1970s to 20 years in 2005 [2]. Although the introduction of Benzimidazole in the early 1980s brought an eminent breakthrough in treatment of AE and CE, surgical therapy is mandatory [28]. Whenever possible complete resection of AE lesions should be performed [2]. Because of the infiltrating growth of parasitic tissue and the potential for metastases principles and rules of tumor surgery should be considered and followed. Unfortunately most patients are inoperable at the time of diagnosis [29]. In the present case a subtotal resection of the right psoas muscle was performed due to the multitude of parasitic lesions and we removed the lumbar vertebrae 1 to 3 and filled them up with bone cement.

Although postoperative Benzimidazole therapy is mandatory in all patients, there is no commonly accepted guideline regarding pre-surgical drug therapy and duration of drug treatment. Some authors suggest that temporary treatment might be sufficient after complete resection [2]. Reuter et al. recommend a Benzimidazole treatment for at least two years since residual parasitic tissue may remain undetected [30]. In case of incomplete surgical resection and in inoperable patients long-term or even life-long Benzimidazole therapy is indicated [2]. Recent studies clearly demonstrated a significant improvement of the 10-year survival rate in non-radically resected patients who received a long term Benzimidazole therapy in comparison to a historic control group [31]. This is in line with the recommendation of Brunetti et al. suggesting lifelong Benzimidazole therapy in non-radically resected patients [2].

Follow-up of infected patients is crucial. This includes imaging in terms of CT and MRI at intervals of 2-3 years and ultrasound at shorter intervals, blood tests with determination of Albendazole blood levels and serological tests. Serological analysis in the follow-up presents a close relationship between clinical status and treatment of patients with AE although interpretation of serological tests in patients under Benzimidazole treatment but without complete resection is more complex [32]. Scheuring et al. demonstrated that anti-Em2- and anti-Em18-antibodies rapidly decreased after complete resection of parasitic lesions and become undetectable [26].

## Conclusion

In summary, primary extrahepatic AE of bone and muscle is an extremely rare disease. The clinical course leading to diagnosis can be long and difficult because of the various and unspecific clinical features. As in the case described psoas muscle abscesses are an important differential diagnosis. Recrudescent reappearance of obvious abscesses in a patient's history despite of the continuous administration of antibiotics and percutaneous drainage for several times has to direct one's attention to the possible presence of Echinococcosis. If a patient is suspected of having Echinococcosis imaging should be completed and serological tests and PCR should be done immediately. Early diagnosis is very important for prognosis and outcome and a multidisciplinary approach of treatment should be followed. Surgery and drug therapy are available options for treatment.

## Competing interests

The authors declare that they have no competing interests.

## Authors' contributions

MN wrote the manuscript and drafted it as well as he performed the review of literature in this context. RB conceived of the study and participated in its design and coordination. GG participated in the design of the case report and review of literature and corrected the first draft. RvER conceived of the study and participated in its design and coordination. CS has read the images and written the images' subtitles. DT as an expert on echinococcosis gave his advice on treatment. CPdC performed the microbiological analysis and gave advice regarding antibiotic treatment. RG approved the case report and review of literature and read the manuscript for corrections. CK conceived of the study and participated in its design and coordination. All authors have read and approved this manuscript.
